# Supplementary material for: Do local and landscape context affect the attractiveness of flower gardens to bees?
Source: PLoS One. 2024 Sep 4;19(9):e0309000. doi: 10.1371/journal.pone.0309000 (PMC11373812; doi:10.1371/journal.pone.0309000)
Supplement: S2 File — (DOCX) [file pone.0309000.s002.docx]

## APPENDIX 2

Supporting materials for “Do local and landscape context affect the attractiveness of flower gardens to pollinators?”

DS Eldridge, A Khalil, JK Moulton, L Russo

**Table S1.** List of plant species used in the experimental research gardens.

| **Asteraceae** | **Fabaceae** | **Lamiaceae** | **Mixed** |
| --- | --- | --- | --- |
| *Helianthus occidentalis* (Riddell) | *Amorpha herbacea* (Walter) | *Conradina verticillata* (Jennison) | *Helianthus occidentalis* |
| *Coreopsis lanceolata* (L.) | *Senna marilandica* (L.) | *Pycnanthemum muticum* (Michx.) | *Senna marilandica* |
| *Eurybia saxicastelli* (J.N. Campbell & M. Medley) | *Baptisia albescens* (Small) | *Lycopus virginicus*(L.) | *Conradina verticillata* |
| *Stokesia laevis* (Hill) | *Lespedeza hirta* (L.) | *Physostegia leptophylla* (Small) | *Baptisia. albescens* |
| *Helianthus hirsutus* (Raf.) | *Baptisia tinctorial* (L.) | *Blephilia subnuda* (Simmers & Kral) | *Coreopsis lanceolata* |
| *Verbesina occidentalis* (Walter) | *Thermopsis villosa* (Walter) | *Collinsonia canadensis* | *Pycnanthemum muticum* |

Table S2. Proportion of developed land use around the surveyed sites. Using GIS, and the NLCD, we classified land use at a 2 km radius around each site.

| **Site** | **Meters** | **Proportion** |
| --- | --- | --- |
| Urban Garden | 2000 | 0.60 |
| Forage Grassland | 2000 | 0.06 |
| Mixed Agriculture | 2000 | 0.05 |
| Forest | 2000 | 0.30 |
| Organic Farm | 2000 | 0.15 |

**Table S3**. Bee species collected in the local and garden surveys.

|  | **Garden Survey** | **Local Survey** |
| --- | --- | --- |
| Andrenidae |  | *Calliopsis andreniformis* |
| Apidae | *Apis mellifera* | *Apis mellifera* |
|  | *Bombus griseocollis* | *Bombus griseocollis* |
|  | *Bombus impatiens* | *Bombus impatiens* |
|  | *Bombus pensylvanicus* | *Bombus pensylvanicus* |
|  | *Ceratina calcarata* | *Ceratina calcarata* |
|  | *Ceratina cockerelli* | *Ceratina cockerelli* |
|  | *Ceratina dupla* | *Ceratina dupla* |
|  | *Ceratina mikmaqi* | *Ceratina mikmaqi* |
|  | *Ceratina strenua* | *Ceratina strenua* |
|  |  | *Epeolus bifasciatus* |
|  | *Holcopasites calliopsidis* | *Holcopasites calliopsidis* |
|  | *Melissodes bimaculatus* | *Melissodes bimaculatus* |
|  |  | *Melissodes communis* |
|  | *Melissodes comptoides* | *Melissodes comptoides* |
|  |  | *Melissodes near boltoniae* |
|  | *Melissodes trinodis* |  |
|  |  | *Xenoglossa (Peponapis) pruinosa* |
|  | *Svastra obliqua* |  |
|  | *Xylocopa virginica* | *Xylocopa virginica* |
| Colletidae |  | *Hylaeus affinis/modestus* |
|  |  | *Hylaeus leptocephalus* |
|  |  | *Hylaeus mesillae* |
| Halictidae | *Agapostemon virescens* | *Agapostemon virescens* |
|  | *Augochlora pura* |  |
|  | *Augochlorella aurata* | *Augochlorella aurata* |
|  | *Augochlorella persimilis* | *Augochlorella persimilis* |
|  | *Augochloropsis metallica* | *Augochloropsis metallica* |
|  | *Halictus confusus* | *Halictus confusus* |
|  | *Halictus poeyi/ligatus* | *Halictus poeyi/ligatus* |
|  | *Halictus paralellus* | *Halictus parallelus* |
|  | *Lasioglossum admirandum* | *Lasioglossum admirandum* |
|  | *Lasioglossum apocyni* | *Lasioglossum apocyni* |
|  | *Lasioglossum callidum* | *Lasioglossum callidum* |
|  | *Lasioglossum coreopsis* |  |
|  | *Lasioglossum coriaceum* |  |
|  |  | *Lasioglossum fattigi* |
|  | *Lasioglossum hitchensi* | *Lasioglossum hitchensi* |
|  |  | *Lasioglossum illinoense* |
|  | *Lasioglossum imitatum* | *Lasioglossum imitatum* |
|  | *Lasioglossum leucocomum* | *Lasioglossum leucocomum* |
|  |  | *Lasioglossum lustrans* |
|  | *Lasioglossum pilosum* | *Lasioglossum pilosum* |
|  | *Lasioglossum pruinosum* | *Lasioglossum pruinosum* |
|  |  | *Lasioglossum simplex* |
|  | *Lasioglossum tegulare* | *Lasioglossum tegulare* |
|  | *Lasioglossum trigeminum* | *Lasioglossum trigeminum* |
|  |  | *Lasioglossum zephyrum* |
|  | *Sphecodes heraclei* |  |
| Megachilidae |  | *Anthidium manicatum* |
|  |  | *Anthidium oblongatum* |
|  | *Coelioxys sayi* | *Coelioxys sayi* |
|  |  | *Hoplitis producta* |
|  | *Megachile brevis* |  |
|  | *Megachile exilis* | *Megachile exilis* |
|  | *Megachile mendica* | *Megachile mendica* |
|  | *Megachile petulans* | *Megachile petulans* |
|  | *Megachile pusilla* | *Megachile pusilla* |
|  | *Megachile rotundata* | *Megachile rotundata* |
|  | *Megachile xylocopoides* |  |

**Table S4.** Results of rarefaction analyses with and without honeybees for the different sites and survey types. We also include the sampling completeness for the sites and survey types.

|  | **Site** | **Diversity** | **Observed** | **Estimator** | **Standard Error** | **Lower Confidence Interval** | **Upper Confidence Interval** |
| --- | --- | --- | --- | --- | --- | --- | --- |
| **Honeybees included** | **Urban Garden** | Shannon diversity | 15.23 | 15.86 | 0.60 | 14.69 | 17.02 |
|  | **Forage Grassland** |  | 30.24 | 32.92 | 1.90 | 29.19 | 36.64 |
|  | **Mixed Agriculture** |  | 26.11 | 28.61 | 1.60 | 25.47 | 31.75 |
|  | **Forest** |  | 8.82 | 9.24 | 0.59 | 8.10 | 10.39 |
|  | **Organic Farm** |  | 12.44 | 13.07 | 0.73 | 11.65 | 14.50 |
|  | **Urban Garden** | Species richness | 79.00 | 122.53 | 23.80 | 79.00 | 169.17 |
|  | **Forage Grassland** |  | 73.00 | 97.16 | 11.11 | 75.39 | 118.93 |
|  | **Mixed Agriculture** |  | 67.00 | 95.75 | 23.23 | 67.00 | 141.29 |
|  | **Forest** |  | 58.00 | 78.14 | 12.97 | 58.00 | 103.56 |
|  | **Organic Farm** |  | 60.00 | 93.30 | 16.60 | 60.77 | 125.83 |
| **Honeybees excluded** | **Urban Garden** | Shannon diversity | 20.95 | 22.20 | 1.00 | 20.24 | 24.15 |
|  | **Forage Grassland** |  | 37.57 | 41.71 | 1.78 | 38.23 | 45.19 |
|  | **Mixed Agriculture** |  | 25.12 | 27.62 | 1.72 | 24.26 | 30.99 |
|  | **Forest** |  | 21.51 | 23.67 | 1.46 | 20.81 | 26.54 |
|  | **Organic Farm** |  | 23.82 | 25.94 | 1.49 | 23.03 | 28.86 |
|  | **Urban Garden** | Species richness | 78.00 | 121.52 | 18.03 | 86.18 | 156.85 |
|  | **Forage Grassland** |  | 72.00 | 96.15 | 14.42 | 72.00 | 124.41 |
|  | **Mixed Agriculture** |  | 66.00 | 94.75 | 11.31 | 72.58 | 116.91 |
|  | **Forest** |  | 57.00 | 77.12 | 12.37 | 57.00 | 101.36 |
|  | **Organic Farm** |  | 59.00 | 92.27 | 15.95 | 61.01 | 123.54 |
| **Honeybees included** | **Local Survey** | Shannon diversity | 16.99 | 17.33 | 0.57 | 16.21 | 18.46 |
|  | **Garden Survey** |  | 28.20 | 29.39 | 1.06 | 27.31 | 31.47 |
|  | **Local Survey** | Species richness | 116.00 | 125.00 | 8.55 | 116.00 | 141.76 |
|  | **Garden Survey** |  | 87.00 | 114.54 | 11.89 | 91.25 | 137.84 |
| **Honeybees excluded** | **Local Survey** | Shannon diversity | 39.78 | 41.17 | 1.08 | 39.05 | 43.28 |
|  | **Garden Survey** |  | 28.25 | 29.55 | 1.15 | 27.29 | 31.81 |
|  | **Local Survey** | Species richness | 115.00 | 124.00 | 7.10 | 115.00 | 137.91 |
|  | **Garden Survey** |  | 86.00 | 113.54 | 15.00 | 86.00 | 142.94 |
|  |  | **Sampling completeness** | |  |  |  |  |
|  | **Urban Garden** | 98.24 |  |  |  |  |  |
|  | **Forage Grassland** | 96.46 |  |  |  |  |  |
|  | **Mixed Agriculture** | 95.77 |  |  |  |  |  |
|  | **Forest** | 97.62 |  |  |  |  |  |
|  | **Organic Farm** | 97.92 |  |  |  |  |  |
|  | **Local Survey** | 99.46 |  |  |  |  |  |
|  | **Garden Survey** | 98.57 |  |  |  |  |  |

**Table S5.** Results of generalized linear mixed effects models using 500 m and 1000 m. Significant effects are bolded.

| **Response** | **Fixed effects** | **Contrasts** | **Family** | **Random effects** | **Observations** | **Estimate** | **z value** | **P value** |
| --- | --- | --- | --- | --- | --- | --- | --- | --- |
| Garden Bee Abundance | **Garden Floral Species Richness** | continuous | Negative Binomial | Round\|Plot | 100 obs, 4 plot types, 5 rounds | 3.82 | 5.9 | **<0.001** |
|  | Local Floral Diversity |  |  |  |  | -1.09 | -1.58 | 0.11 |
|  | Development (1km) |  |  |  |  | 0.02 | 1.87 | 0.06 |
| Local Bee Abundance | **Local Floral Display** | continuous | Negative Binomial | Round\|Plot | 100 obs, 4 plot types, 5 rounds | 1.32 | 2.1 | **0.04** |
|  | Development (1km) |  |  |  |  | 0.01 | 1.55 | 0.12 |
| Garden Bee Species Richness | **Garden Floral Display** | continuous | Negative Binomial | Round\|Plot | 100 obs, 4 plot types, 5 rounds | 1.58 | 4.87 | **<0.001** |
|  | Local Floral Display |  |  |  |  | -0.51 | -1.21 | 0.23 |
|  | **Development (1km)** |  |  |  |  | 0.01 | 2.09 | **0.04** |
| Local Bee Species Richness | **Local Floral Display** | continuous | Negative Binomial | Round\|Plot | 100 obs, 4 plot types, 5 rounds | 0.85 | 3.04 | **0.002** |
|  | Development (1km) |  |  |  |  | 0.003 | 0.66 | 0.51 |
| Garden Bee Abundance | **Garden Floral Species Richness** | continuous | Negative Binomial | Round\|Plot | 100 obs, 4 plot types, 5 rounds | 3.68 | 5.68 | **<0.001** |
|  | Local Diversity |  |  |  |  | -0.74 | -1.05 | 0.29 |
|  | Development (500 m) | |  |  |  | 0.01 | 1.22 | 0.22 |
| Local Bee Abundance | **Local Floral Display** | continuous | Negative Binomial | Round\|Plot | 100 obs, 4 plot types, 5 rounds | 1.79 | 2.84 | **0.005** |
|  | Development (500 m) | |  |  |  | 0.005 | 0.63 | 0.53 |
| Garden Bee Species Richness | **Garden Floral Display** | continuous | Negative Binomial | Round\|Plot | 100 obs, 4 plot types, 5 rounds | 1.52 | 4.62 | **<0.001** |
|  | Local Floral Display |  |  |  |  | -0.23 | -0.55 | 0.58 |
|  | Development (500 m) | |  |  |  | 0.007 | 1.28 | 0.2 |
| Local Bee Species Richness | **Local Floral Display** | continuous | Negative Binomial | Round\|Plot | 100 obs, 4 plot types, 5 rounds | 0.94 | 3.31 | **0.001** |
|  | Development (500 m) | |  |  |  | 0.001 | 0.25 | 0.81 |

**Table S6.** Results of indicator species tests for significant species associations.

| **Test** | **Group** | **Indicator species** | **R** | **P value** |
| --- | --- | --- | --- | --- |
| **Sites** | Urban Garden | *Bombus pensylvanicus* | 0.76 | 0.001 |
|  |  | *Megachile pusilla* | 0.70 | 0.002 |
|  |  | *Bombus impatiens* | 0.69 | 0.001 |
|  |  | *Xylocopa virginica* | 0.67 | 0.001 |
|  |  | *Lasioglossum apocyni* | 0.64 | 0.001 |
|  |  | *Megachile rotundata* | 0.60 | 0.002 |
|  |  | *Megachile mendica* | 0.58 | 0.006 |
|  |  | *Bombus griseocollis* | 0.56 | 0.002 |
|  |  | *Anthidium manicatum* | 0.46 | 0.031 |
|  |  | *Agapostemon virescens* | 0.45 | 0.045 |
|  | Forage Grassland | *Ceratina dupla* | 0.48 | 0.043 |
|  | Forest | *Lasioglossum lustrans* | 0.49 | 0.005 |
|  | Organic Farm | *Lasioglossum zephyrum* | 0.54 | 0.027 |
|  |  | *Lasioglossum admirandum* | 0.47 | 0.045 |
|  |  | *Lasioglossum tegulare* | 0.45 | 0.015 |
| **Plot Types** | Asteraceae | *Halictus ligatus/poeyi* | 0.54 | 0.003 |
| **Survey method** | Local Survey | *Apis mellifera* | 0.52 | 0.001 |
|  |  | *Lasioglossum hitchensi* | 0.48 | 0.003 |
|  |  | *Lasioglossum callidum* | 0.47 | 0.004 |
|  |  | *Lasioglossum imitatum* | 0.43 | 0.002 |
|  |  | *Calliopsis andreniformis* | 0.39 | 0.001 |
|  |  | *Lasioglossum trigeminum* | 0.35 | 0.045 |
|  |  | *Lasioglossum apocyni* | 0.32 | 0.038 |

**Figure S1**. Pie charts of relative abundance of different insect specimens collected.

**
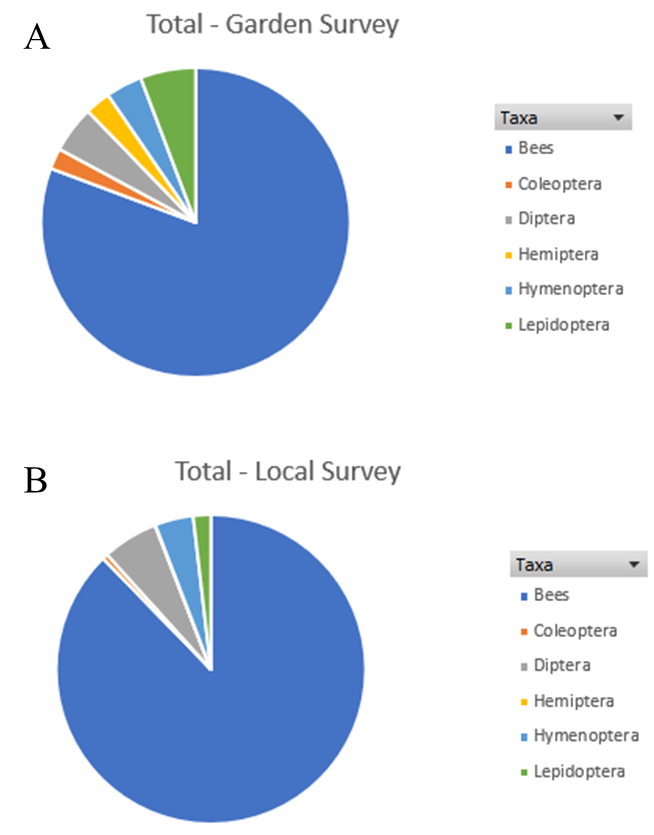
**

**Figure S1.** Pie chart showing how many bee specimens (dark blue) were collected compared to all non-bee specimens (other colors) collected during the garden plots (A) and local (B) surveys.

**Figure S2.** Rarefaction analyses with and without honeybees, along with graphs of sample coverage.


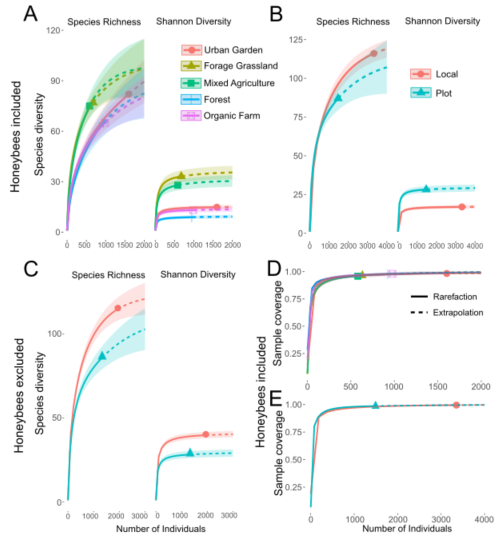


Figure S2. Comparing rarefaction analyses of the species richness and Shannon diversity of the five different land-use sites (red = Urban Garden, yellow = Forage Grassland, green = Mixed Agriculture, blue = Forest, and pink = Organic Farm) with honeybees (A). There are also rarefaction comparisons of the landscape and plot surveys with (B) and without (C) honeybees. Removing honeybees from the analysis shows how this abundant species affects the evenness of the bee community. The solid lines indicate the interpolated (observed) diversity at the different sites, while the dotted lines indicate the extrapolated (predicted) diversity. We also include the sample coverage for the different sites (D) and surveys (E).
