# Supplementary material for: Do local and landscape context affect the attractiveness of flower gardens to bees?
Source: PLoS One. 2024 Sep 4;19(9):e0309000. doi: 10.1371/journal.pone.0309000 (PMC11373812; doi:10.1371/journal.pone.0309000)
Supplement: Attachment — Submitted filename: PONE-D-24-09528_reviewer.pdf [file pone.0309000.s003.pdf]

PLOS ONE

Do local and landscape context affect the attractiveness of flower gardens to bees?

--Manuscript Draft--

**Manuscript Number:**	PONE-D-24-09528
**Article Type:**	Research Article
**Full Title:**	Do local and landscape context affect the attractiveness of flower gardens to bees?
**Short Title:**	Effects of context on pollinator garden attractiveness
**Corresponding Author:**	Laura Russo<br>University of Tennessee<br>Knoxville, TN UNITED STATES
**Keywords:**	Floral resources, land use, urban ecology, pollinator conservation, biodiversity
**Abstract:**	Planting floral resources is a common strategy for increasing the abundance and diversity of beneficial insects in human-modified systems. However, the context of the local area and surrounding landscape may affect the attractiveness of these floral resource provisioning plots. We compared the relative effects of local flowering plant diversity and abundance and surrounding urban land-use on the attractiveness of common gardens to bees in eastern Tennessee, USA. We planted four common garden plots at each of five different landscapes representing a variety of surrounding land use: 1) Urban Garden, 2) Forage Grassland, 3) Mixed Agriculture, 4) Forest, and 5) Organic Farm. The common garden plots had fixed plant communities, replicated at all of the different sites. We concurrently sampled bees in the garden plots and in a 50 m radius (local area) around the garden plots. We found that floral display in the local area did not affect bee abundance or species richness in the garden plots, but that bee abundance in the gardens was associated with the flower species richness in the local area. Although there was a significant positive association between developed land use in a 2 km radius around the gardens and bee abundance and species richness in the gardens, the effect was small, and there was no relationship between land use and bee abundance or species richness in the local area. There were significant differences in the composition of the bee community between the local area and garden plots, but the largest determinants of bee community composition and diversity in the gardens were variation in the garden plant species and floral display size. This finding is promising for anyone wishing to promote pollinator populations by providing more floral resources.
**Order of Authors:**	Devon S Eldridge, MS
	Amani Khalil, MS
	John K Moulton, Phd
	Laura Russo
**Additional Information:**	
**Question**	**Response**
**Financial Disclosure**<br><br>Enter a financial disclosure statement that describes the sources of funding for the work included in this submission. Review the submission guidelines for detailed requirements. View published research articles from *PLOS ONE* for specific examples.<br><br>This statement is required for submission	Yes

Powered by Editorial Manager® and ProduXion Manager® from Aries Systems Corporation

and **will appear in the published article** if the submission is accepted. Please make sure it is accurate.<br><br>**Funded studies**<br>Enter a statement with the following details:<br>• Initials of the authors who received each award<br>• Grant numbers awarded to each author<br>• The full name of each funder<br>• URL of each funder website<br>• Did the sponsors or funders play any role in the study design, data collection and analysis, decision to publish, or preparation of the manuscript?<br><br>Did you receive funding for this work?	
Please add funding details.<br>as follow-up to "**Financial Disclosure**<br><br>Enter a financial disclosure statement that describes the sources of funding for the work included in this submission. Review the submission guidelines for detailed requirements. View published research articles from *PLOS ONE* for specific examples.<br><br>This statement is required for submission and **will appear in the published article** if the submission is accepted. Please make sure it is accurate.<br><br>**Funded studies**<br>Enter a statement with the following details:<br>• Initials of the authors who received each award<br>• Grant numbers awarded to each author<br>• The full name of each funder<br>• URL of each funder website<br>• Did the sponsors or funders play any role in the study design, data collection and analysis, decision to publish, or preparation of the manuscript?<br><br>Did you receive funding for this work?"	LR received funding from Bayer in 2019 ($5000 Feed A Bee)

Please select the country of your main research funder (please select carefully as in some cases this is used in fee calculation).<br>as follow-up to "**Financial Disclosure**<br><br>Enter a financial disclosure statement that describes the sources of funding for the work included in this submission. Review the submission guidelines for detailed requirements. View published research articles from *PLOS ONE* for specific examples.<br><br>This statement is required for submission and **will appear in the published article** if the submission is accepted. Please make sure it is accurate.<br><br>**Funded studies**<br>Enter a statement with the following details:<br>• Initials of the authors who received each award<br>• Grant numbers awarded to each author<br>• The full name of each funder<br>• URL of each funder website<br>• Did the sponsors or funders play any role in the study design, data collection and analysis, decision to publish, or preparation of the manuscript?<br><br>Did you receive funding for this work?"	UNITED STATES - US
**Competing Interests**<br><br>Use the instructions below to enter a competing interest statement for this submission. On behalf of all authors, disclose any competing interests that could be perceived to bias this work—acknowledging all financial support and any other relevant financial or non-financial competing interests.<br><br>This statement is required for submission and **will appear in the published article** if the submission is accepted. Please make sure it is accurate and that any funding	The authors have declared that no competing interests exist.

sources listed in your Funding Information later in the submission form are also declared in your Financial Disclosure statement.<br><br>View published research articles from *PLOS ONE* for specific examples.<br><br>**NO authors have competing interests**<br><br>Enter: *The authors have declared that no competing interests exist.*<br><br>**Authors with competing interests**<br><br>Enter competing interest details beginning with this statement:<br><br>*I have read the journal's policy and the authors of this manuscript have the following competing interests: [insert competing interests here]*<br><br>* typeset	
**Ethics Statement**<br><br>Enter an ethics statement for this submission. This statement is required if the study involved:<br><br>• Human participants<br>• Human specimens or tissue<br>• Vertebrate animals or cephalopods<br>• Vertebrate embryos or tissues<br>• Field research<br><br>Write "N/A" if the submission does not require an ethics statement.<br><br>General guidance is provided below. Consult the submission guidelines for detailed instructions. **Make sure that all information entered here is included in the Methods section of the manuscript.**	We had permission to conduct field research at the University of Tennessee Research and Education Centers.

**Format for specific study types**<br><br>**Human Subject Research (involving human participants and/or tissue)**<br>• Give the name of the institutional review board or ethics committee that approved the study<br>• Include the approval number and/or a statement indicating approval of this research<br>• Indicate the form of consent obtained (written/oral) or the reason that consent was not obtained (e.g. the data were analyzed anonymously)<br><br>**Animal Research (involving vertebrate animals, embryos or tissues)**<br>• Provide the name of the Institutional Animal Care and Use Committee (IACUC) or other relevant ethics board that reviewed the study protocol, and indicate whether they approved this research or granted a formal waiver of ethical approval<br>• Include an approval number if one was obtained<br>• If the study involved *non-human primates*, add *additional details* about animal welfare and steps taken to ameliorate suffering<br>• If anesthesia, euthanasia, or any kind of animal sacrifice is part of the study, include briefly which substances and/or methods were applied<br><br>**Field Research**<br><br>Include the following details if this study involves the collection of plant, animal, or other materials from a natural setting:<br>• Field permit number<br>• Name of the institution or relevant body that granted permission	
**Data Availability**<br><br>Authors are required to make all data underlying the findings described fully available, without restriction, and from the time of publication. PLOS allows rare exceptions to address legal and ethical concerns. See the PLOS Data Policy and FAQ for detailed information.	Yes - all data are fully available without restriction

A Data Availability Statement describing where the data can be found is required at submission. Your answers to this question constitute the Data Availability Statement and **will be published in the article**, if accepted.<br><br>**Important:** Stating 'data available on request from the author' is not sufficient. If your data are only available upon request, select 'No' for the first question and explain your exceptional situation in the text box.<br><br>Do the authors confirm that all data underlying the findings described in their manuscript are fully available without restriction?	
**Describe where the data may be found in full sentences. If you are copying our sample text, replace any instances of XXX with the appropriate details.**<br><br>• If the data are **held or will be held in a public repository**, include URLs, accession numbers or DOIs. If this information will only be available after acceptance, indicate this by ticking the box below. For example: *All XXX files are available from the XXX database (accession number(s) XXX, XXX.).*<br>• If the data are all contained **within the manuscript and/or Supporting Information files**, enter the following: *All relevant data are within the manuscript and its Supporting Information files.*<br>• If neither of these applies but you are able to provide **details of access elsewhere**, with or without limitations, please do so. For example:<br><br>*Data cannot be shared publicly because of [XXX]. Data are available from the XXX Institutional Data Access / Ethics Committee (contact via XXX) for researchers who meet the criteria for access to confidential data.*<br><br>*The data underlying the results presented in the study are available from (include the name of the third party*	All relevant data are within the manuscript and its Supporting Information files.

Powered by Editorial Manager® and ProduXion Manager® from Aries Systems Corporation

and contact information or URL).<br>• This text is appropriate if the data are owned by a third party and authors do not have permission to share the data.<br><br>* typeset	
Additional data availability information:	

Manuscript

Click here to access/download;Manuscript;Eldridge_etal__PLoSONE.docx

Full Title: Do local and landscape context affect the attractiveness of flower gardens to bees?

Short title: Effects of context on pollinator garden attractiveness

Devon S. Eldridge[1], deldrid5@vols.utk.edu

Amani Khalil[1], akhalil1@vols.utk.edu

John K. Moulton[2], jmoulton@utk.edu

Laura Russo[1*], lrusso@utk.edu

[1]Department of Ecology & Evolutionary Biology, University of Tennessee, Knoxville, TN USA 37996

[2] Department of Entomology & Plant Pathology, University of Tennessee, Knoxville, TN USA 37996

*** = corresponding author**

Journal: PLoS ONE

Paper type: Original Article

Word Count: Abstract 283, Main Text 3,708, 51 References, 1 Table, 4 Figures, Supplemental Materials (4 Tables, 3 Figures)

Author contributions. DE and LR contributed to the conceptualization, data curation, formal analysis, methodology, validation, and visualization of the project. DE and MK collected and curated the data and conducted investigation. LR was in charge of project

administration and KM contributed to supervision, validation, and data curation. DE wrote the original draft of the manuscript and all authors contributed substantially to editing and revising the manuscript.

Data Availability Statement. All relevant data are within the manuscript and its Supporting Information files.

Declaration of Competing Interest. None.

Funding Statement. Funding was provided by Bayer’s Feed-A-Bee grant to LR in 2019.

Abstract. Planting floral resources is a common strategy for increasing the abundance and diversity of beneficial insects in human-modified systems. However, the context of the local area and surrounding landscape may affect the attractiveness of these floral resource provisioning plots. We compared the relative effects of local flowering plant diversity and abundance and surrounding urban land-use on the attractiveness of common gardens to bees in eastern Tennessee, USA. We planted four common garden plots at each of five different landscapes representing a variety of surrounding land use: 1) Urban Garden, 2) Forage Grassland, 3) Mixed Agriculture, 4) Forest, and 5) Organic Farm. The common garden plots had fixed plant communities, replicated at all of the different sites. We concurrently sampled bees in the garden plots and in a 50 m radius (local area) around the garden plots. We found that floral display in the local area did not affect bee abundance or species richness in the garden plots, but that bee abundance in the gardens was associated with the flower species richness in the local area. Although there was a significant positive association between developed land use in a 2 km radius around the gardens and bee abundance and species richness in the gardens, the effect was small, and there was no relationship between land use and bee abundance or species richness in the local area. There were significant differences in the composition of the bee community between the local area and garden plots, but the largest determinants of bee community composition and diversity in the gardens were variation in the garden plant species and floral display size. This finding is promising for anyone wishing to promote pollinator populations by providing more floral resources.

Key Words. Floral resources, land use, urban ecology, pollinator conservation, biodiversity

1. Introduction.

As the human population grows, so does the global production of food, both in terms of the extent and intensity of agricultural production (1). Many crop species rely on insect pollination, and land-use intensification has been shown to reduce both pollinating insect species richness and abundance (2,3). For example, while native bees can provide sufficient pollination services on watermelon farms, continued agricultural intensification drastically decreases unmanaged pollination services (4–6). In fact, land use changes have been implicated as a primary driver of insect declines worldwide (7).

The extent of developed landscapes is accelerating globally and although they are sometimes considered pesticide-free refuges for bees (8), urban areas can have negative effects on pollinating insects (9,10). Urban land-use is unique in the way it alters landscapes, increasing the prevalence of non-native species and significantly altering abiotic factors such as temperature and the extent of impervious surfaces (11). As a way to ameliorate the impact of such intensive land-use on beneficial arthropods, those interested in protecting pollinating insects recommend floral resource provisioning strips (8). These strips of floral resources, essentially flower gardens, have been shown to increase pollinator abundance and diversity in intensively managed systems (12–14), but their attractiveness to pollinators also seems to vary with landscape context (15–17).

The attractiveness of flowering gardens may be affected by local factors, such as floral resources in the immediate area around the plot (15), or broader factors, such as land-use on a wider scale (17). For example, at the local scale, the relative attractiveness of any given plant species might be affected by neighboring flowering plants (18). There

is evidence for both a magnet effect, where having an attractive neighbor increases visitation to a plant (19), and a competitive effect, where having an attractive neighbor decreases visitation to a plant (20). These two effects may in part be moderated by the availability of resources at a broader scale.

Land-use has been repeatedly shown to have effects on pollinating insects (21,22). For example, agricultural land use was shown to decrease phylodiversity of bee communities (3), and urban land use was shown to have a negative effect on gene flow in *Bombus vosnesenskii* (23) and to change the microbiome in *Ceratina calcarata* (24). While the negative impacts of agricultural land use seem to depend on the intensity and extent of the management (25), urban land use does not always have clear negative effects on pollinating insects, and the impact can depend on taxa and traits (26,27). It is possible that local floral resources, such as urban gardens, may be mediating the impact of urban land use (28).

Our goal was to determine whether the attractiveness of flowering garden plots was affected by local or landscape factors, or an interaction between them. We established four common garden plots at each of five different sites varying in both local flowering resources and land-use at a broader scale. Our hypotheses were that: 1) the abundance and diversity of bees visiting flowers in the garden plots would be mostly driven by the abundance and diversity of local flowering resources, 2) land-use at a broader scale would interact with local floral availability to affect bee abundance and richness in the gardens, and 3) the bee community in the gardens would be a subset of the surrounding bee community.

Materials and Methods.

Experimental Design

We established garden plots with a fixed community of native perennial plants in eastern Tennessee, USA. Each of the gardens contained four individuals of each of six perennial wildflower species native to Tennessee. In each of five sites, we planted four 3m x 2m garden plots: 1) six species of the plant family Asteraceae (A), 2) six species of Fabaceae (F), 3) six species of Lamiaceae (L), and 4) a mixed plot two species of each of the aforementioned families (mixed plot, M) (Fig. [illegible]ig. S1). At each site, the plots were separated between 15m – 50m. We chose the plant composition (a total of 18 species) based on commercially available, pollinator-attractive plant species native to the region and sourced them from a local native plant nursery (Overhill Gardens, Vonore, TN, Table S1).

All of the gardens were established following the same methodology at five separate sites: 1) Urban Garden, 2) Forage Grassland, 3) Mixed Agriculture, 4) Forest, and 5) Organic Farm. These sites were selected because they represented a variety of local and landscape types, with land-use at a 2 km radius varying from primarily urban (Urban Garden) to primarily agricultural (Forage Grassland). The community of flowering plants immediately surrounding the gardens varied from frequently mown (Forage Grassland), to a high diversity of native (Forest) and ornamental (Urban Garden) flowering beds. Both the Mixed Agriculture and Organic Farm sites comprised a mixed community of taller grasses and some common non-native flowering weeds, that were

mown twice a summer, alongside a mixture of research crops including blueberries, apples, and switchgrass (Mixed Agriculture) and lettuce, squash, and mint (Organic Farm). The fixed community of plants within the gardens allowed us to isolate the effects of local and landscape factors on changes in the flower-visiting insect communities.

Garden Plot Surveys

Each garden plot was visited weekly from July 13th – August 17th, 2020, or five complete rounds of sampling each plot in the study. The amount of time spent sampling in each garden varied depending on the number of plant species in flower (5 min per plant species in bloom, or up to 30 min per garden).

We collected all insects that contacted the reproductive parts of the inflorescences within the garden plots during standardized surveys. These weekly surveys involved sampling each plant species in bloom in each plot at a given site for five minutes, using a hand-held vacuum aspirator from BioQuip (Rancho Dominquez, CA). During each collection event, we also counted the number of inflorescences of each plant species and used the number of inflorescences per plot as a measure of overall floral display. We kept track of this to determine how the background floral display within the plot affected its attractiveness to pollinating insects (29–31).

Local Survey

We paired the plot surveys with surveys in the local area immediately around each plot. These local surveys were paired in time (July 13th – August 17th 2020) and space with the plots. An insect net was used to capture insects that contacted the reproductive parts of flowers within a 50m radius of each research plot once a week, for a total of five

complete surveys (Fig. 1). These local surveys were 10 minutes each, allowing the collector to walk the entirety of the area within the 50m radius. Honeybees (*Apis mellif*[illegible] L) and carpenter bees (*Xylocopa virginica* L) were counted and recorded but not captured, as they could be identified on sight.

Flowering plant diversity and floral abundance were surveyed before each pollinator survey was performed. We used a visual ranking method within the 50m radius around each research plot in the following manner. First, we ranked the flowering plant diversity on a scale from 1–10, where 1 was the least diverse survey area, and 10 was the most diverse survey area. Then we ranked floral abundance on a scale from 1–10, where 1 was the smallest, and 10 was the largest floral abundance within a survey area. Such ranking methods have been shown to be consistent with other vegetation measures and respond consistently to treatments [illegible]). The same surveyor ranked diversity and floral display each time, according to their own perception. These measures allowed us to qualitatively compare the survey areas to one other.

Insect processing

Insects collected from both garden and local surveys were frozen, then pinned, labeled, identified, and databased. We focused our analysis on bee specimens (members of the superfamily Apoidea). Bees were identified to genus and species where possible, using the Discover Life interactive key (33). Bee identifications were then verified by Sam Droege (USG[illegible] Specimens are vouchered at the University of Tennessee.

Landscape Analysis

To classify the landscape around our research sites, we used ArcGIS Pro and the US National Land Cover Database (NLCD) (https://www.mrlc.gov/) (34). We classified land cover at 2000 m around the center of each site (Fig 1). Our goal in using this buffer radius was to determine how the broader landscape affected bee diversity and abundance in the gardens (35,36). In our analysis, we detected 14 land cover classifications that we aggregated into four general land-use types: water (Open Water), developed (Developed High Intensity, Developed Low Intensity, Developed Medium Intensity, Developed Open Space), agriculture (Cultivated Crops, Grassland/Herbaceous, Pasture/Hay), and semi-natural (Deciduous Forest, Emergent Herbaceous Wetlands, Evergreen Forest, Mixed Forest, Shrub/Scrub, Woody Wetlands) (Table S1). The NLCD defined developed land as constructed materials and impervious surfaces such as commercial and residential housing, roadways, and lawn grasses. Areas classified as water included open water and areas with minimal soil and vegetation. Semi-natural land use included different forest types, wetlands, shrubland, and non-grassland herbaceous land cover. Agricultural land use comprised of pastureland, cultivated crops, or grasslands. We hypothesized that landscape context would interact with local floral resources to have an effect on bee diversity or abundance; specifically, that landscapes that increased bee diversity and abundance in the local surveys would also increase bee diversity and abundance in the garden plots.

Data Analysis

All data analyses were conducted in R (37). First, we compared the bee collections among the sites and surveys (garden or local). We used feature scaling to standardize the

measures of floral display and diversity in the gardens and local area on a scale of 0 – 1. We compared the average number of bees collected per minute sampling, and the relationship between bee abundance and the scaled floral display for both survey types. We used a rarefaction analysis to test for differences in bee species richness and Shannon diversity index among the sites and surveys.

To test our first hypothesis, we then used generalized linear mixed effects models (GLMMs) using the function *lmer* in the package "lme4" (38) to test the effect of the scaled floral abundance and flower richness in the garden and local area, and developed land use at a 2 km radius on bee abundance and species richness in the gardens, with sampling date nested in site as the random effect. We also used GLMMs to test whether the abundance and species richness of bees in the area around the gardens was affected by land use or local floral display and species richness. To test our second hypothesis about whether land use interacted with local floral display and flower richness, we also tested for interactions between the land use effect and the other fixed effects. We removed non-significant interaction terms from the final models. For all models, we used a Gaussian distribution and tested for the normality of the residuals using a Shapiro-Wilk test. Where models failed this normality test, we log-transformed the response variable.

To test our third hypothesis, we used non-metric multidimensional scaling (NMDS) to compare the bee community composition among the sites and surveys (39). NMDS is an ordination technique that allows for visualization of multivariate responses to treatments. In our case, we were looking for overlap, or non-overlap in the community structure of the bees. We used the function *anosim* in the package "vegan" (39) to test for

significant differences in the bee community composition. We used 99 permutations and Bray-Curtis dissimilarity in this test. To determine which species were driving differences, we used the *multipatt* function in the package "indicspecies" (40) when the groups differed significantly.

3. Results.

Garden Plot Surveys

We collected 1,470 specimens during 20.83 hours of sampling in the garden plots (34). Most of the specimens (1,186 or 81%) were bees (Halictidae, Megachilidae, and Apidae) (Fig. S2A), of which 137 (9%) were honeybees (*Apis mellifera*). A total of 44 bee species were collected. Halictidae (791 specimens) was the most abundant bee family collected within the gardens during the sampling period. Apidae followed as the second most abundant bee family with 356 specimens. The largest number of specimens were collected from plots at the Forage Grassland (379) and the Organic Farm (318).

On average, 2.4 $\pm$ 0.13 (standard error) plant species were in bloom per plot during the surveys. We observed an average of 369.21 $\pm$ 39.8 inflorescences per plot per survey. We used feature scaling to standardize these measures to a 0 – 1 scale to compare to the plant diversity and floral abundance of the local surveys.

Local Surveys

We collected or observed 3,324 flower-visiting insects during 16.67 hours of sampling the local areas surrounding the plots. Most of the specimens (2,917 or 88%) were bees (Halictidae, Megachilidae, Colletidae, Andrenidae, and Apidae) (Fig. S2B); 1,380 (42%)

were *A. mellifera* (Fig. S2). We collected 52 species of bees (Table S3). We collected six species of bees not found in the plot surveys: *Melissodes communis* Cresson, *Xenoglossa* (*Peponapis*) *pruinosa* (Say), *Hylaeus leptocephalus* (Morawitz), *Lasioglossum fattigi* (Mitchell), *Lasioglossum simplex* (Robertson), and *Hoplitis producta* Cresson. *Lasioglossum fattigi* and *L. simplex* were both new occurrence records for the state of Tennessee (*pers. comm.*, S. Droege, J. Ascher).

The average ranked floral abundance in the local survey was 4.25 $\pm$ 0.27 (standard error) and the average ranked plant diversity was 4.17 $\pm$ 0.29. We used feature scaling to standardize these ranked floral abundance and plant diversity measures from 0 – 1.

Comparing garden and local surveys

The Mixed Agriculture site had the highest bee abundance among the garden surveys, while the Urban Garden site had the highest abundance among the local surveys (Fig. 2A). The local surveys found a higher bee abundance per minute spent sampling than the garden plots at four of the five sites (Fig. 2A). The scaled floral abundance had a significant positive effect on bee abundance in both local and garden surveys (Fig. 2B). The rarefaction analysis showed high sample coverage for the sites we sampled (Fig. 2C). The Forage Grassland and Mixed Agriculture sites both had significantly higher bee species richness and Shannon diversity than the other sites (Fig. 2C). We also compared the local and garden surveys with and without honeybees (Fig. S3) and found that they did not differ in species richness, but that the gardens had a higher Shannon diversity than the local surveys due to a greater species evenness. This difference was probably driven

by the dominance of honeybees in the local surveys because, after removing honeybees from the rarefaction analysis, we found that the local surveys had a higher Shannon diversity than the plots (Fig. S3).

We did not find any significant interactions between developed land use at a 2 km radius and any of the other fixed effects and thus removed the interaction terms from the models. The abundance of bees in the gardens was significantly associated with the floral display and the flower species richness in the gardens, along with local flower species richness and developed land use at a 2 km radius (Table 1, Fig. 3A, B, C, D). However, the effects of the latter two were much smaller in magnitude than the effects of the flowers in the gardens themselves. Bee abundance in the gardens was not associated with local floral display. Similarly, the species richness of bees in the gardens was significantly associated with flower species richness in the gardens and developed land use at a 2 km radius, but not floral display in the gardens, or local floral display or species richness (Table 1, Fig. 3E, F). Bee abundance and species richness in the local surveys were only associated with local floral display (Table 1, Fig. 3G, H).

The NMDS ordination plots (Fig. 4) showed community overlap between the sites, plot types, and surveys. All three groups differed significantly from one another, with low R values, suggesting little overlap in community composition (sites R = 0.19, p = 0.01; plot types R = 0.08, p = 0.04, survey types R = 0.19, p = 0.01). Our indicator species analysis suggested that the sites had the largest number of species with significant differences (Table S4). The Urban Garden site had the largest number of significantly associated bee species (*Bombus impatiens, B. pensylvanicus, B. griseocollis, Megachile*

pusilla, M. rotundata, M. mendica, Xylocopa virginica, Lasioglossum apocyni, Anthidium manicatum, and *Agapostemon virescens*), followed by the Organic Farm (*Lasioglossum zephyrum, L. admirandum, L. tegulare*), the Forage Grassland (*Ceratina dupla*), and the Forest (*Lasioglossum lustrans*) (Table S4). For the plot types, the only significantly associated bee species was *Halictus ligatus/poeyi*, which was significantly associated with the Asteraceae plots (Table S4). Between the two survey methods, there were seven species significantly associated with the local surveys (*Apis mellifera, Lasioglossum hitchensi, L. callidum, L. imitatum, L. trigeminum, L. apocyni*, and *Calliopsis andreniformis*) and none associated with the gardens (Table S4).

Discussion.

In our study of the relative effects of local and landscape characteristics on the attractiveness of flowers to visiting bees, we found strong positive relationships between floral display and bee abundance and species richness, but also that the effect was mostly constrained to the sample area, whether it be the common garden itself or the local area. Similar relationships have been previously documented in agricultural ecosystems (41,42) and strong background effects of floral abundance on pollinator visitation are well-documented (31,43). We observed the highest local plant diversity and floral abundance in the site managed as a public garden (Urban Garden site). This site was well manicured, with many flower beds for display. At the same time, this high floral display and diversity in the local area did not significantly increase the abundance or species richness of the bees in our garden plots. Our first hypothesis, that local floral display and

diversity would be the primary driver of bee abundance and species richness in the garden plots, was therefore not supported by our results. This contrasts with other studies that have shown that the surrounding floral resources and landscape context can significantly affect bee abundance and diversity in wildflower gardens (15,44).

Our second hypothesis was that the surrounding landscape would interact with local floral resources to drive bee abundance and species richness in the gardens. However, we found no significant interactions between our land use variable and the other factors in our analysis. While developed land use at a 2 km radius did have a significant positive effect on bee abundance and species richness in the gardens, the effect size was orders of magnitude smaller than the effect of the floral display and diversity in the gardens themselves. Moreover, developed land use had no effect on the bee abundance or species richness in the local surveys. Thus, our second hypothesis was also not supported. Overall, local factors seemed to be the major drivers of the bee community, at least in this study. It is possible that other land-uses, such as agricultural land or natural areas, may have had an undetected influence on the pollinator communities in these systems. Further, our sites were in patchy landscapes and this patchy, heterogenous habitat may influence bee species composition and abundance (45,46). Measuring landscape heterogeneity and patch structure in the landscape might show different patterns.

It is also possible that more extreme landscapes would have shown stronger effects. Our sites varied between roughly 5% and 60% developed land-use at a 2 km radius, whereas one previous study found non-linear effects on homogenization in

Lepidoptera above 60% agricultural land-use (47). Our findings might therefore not be representative of more homogenous landscapes. However, even if this were true, we would at least expect to see some difference between the two extreme ends of our gradient. Instead, we saw a mild positive effect of developed land use on overall bee abundance and species richness in the gardens. Previous studies similarly found multiple non-linearities and significant variation in patterns of community similarity (48).

Our third hypothesis was that the bee community in the research gardens would be a subset of the local bee community. Overall, the sites and surveys had relatively similar total bee species richness, but the evenness of the species varied, leading to a greater difference in the Shannon diversity index. For example, although there were not significant differences in the total bee species richness of the two surveys, the garden survey had a higher Shannon diversity until we removed honeybees from the analysis. Proportionally, far fewer honeybees were collected in the gardens (~9%) than in the surrounding local surveys (~53%), which may have been due to the plant species composition of the plots relative to the local area. Eusocial bee species may be capable of competitively excluding solitary bee species (49), but may also exhibit distinct foraging preferences (50). Although on a per minute sampling basis, the local surveys collected a higher abundance of bees, from a bee community composition perspective, the differences among sites, plot types, and survey types were relatively minor. Similarly, though these groups differed significantly, there substantial overlap in the community composition. We were able to identify significant indicator species among the different groups, and found that the Urban Garden and the local surveys had the largest number of

significantly associated species, while there were no bee species significantly associated with the research gardens. From these results, we find mixed support for the hypothesis that the bees in the garden plots were a subset of the surrounding bee community.

5. Conclusions.

Our study provides promising findings for anyone that wishes to promote healthy pollinator communities. Along with other research, this study supports strategies for promoting pollinators by increasing plant diversity and floral display (51). Our findings did not show support for our hypotheses that bee abundance and species richness in experimental gardens would be driven by a mixture of local and landscape effects. Instead, we saw that these gardens attracted a diverse bee community largely through the size of their floral display and the identity of the blooming plants within them. We found minor effects of developed land use but no large effects across a range of land use. We show that local floral resources are stronger predictors of bee abundance than surrounding land-use and that managed landscapes can maintain high abundances of bees. Whether undisturbed or managed, landowners have the power to promote their local pollinator communities by conserving existing natural patches or increasing the availability of high-quality floral resources.

Acknowledgements. We would like to thank the University of Tennessee (UT), the UT Institute of Agriculture, the UT Gardens, and the UT Ag Research and Education

Centers, including the Plateau AgResearch Center, the Organic Crops Unit, and the Forest Research and Education Center, for allowing us to do research on their lands, especially K. Hoyt, B. Simpson, W. Hitch, H. Jones, J. Newburn, and W. Lively. We would also like to thank D. Matheson, S. Collins, and A. Murray for field assistance and N. Oldham for assisting with hoverfly identification. Thanks also go to S. Droege for lending his expertise in bee identification. We are also grateful to Bayer, for the Feed-A-Bee grant that funded our Feed-A-Bee research plots. Thank you to the Department of Ecology & Evolutionary Biology at the University of Tennessee for the support of this project.

REFERENCES

1. Sanderson EW, Jaiteh M, Levy MA, Redford KH, Wannebo AV, Woolmer G. The human footprint and the last of the wild: The human footprint is a global map of human influence on the land surface, which suggests that human beings are stewards of nature, whether we like it or not. Bioscience. 2002;52(10):891–904.
2. Flynn DFB, Gogol-Prokurat M, Nogeire T, Molinari N, Richers BT, Lin BB, et al. Loss of functional diversity under land use intensification across multiple taxa. Ecology Letters. 2009;12(1):22–33.
3. Grab H, Branstetter MG, Amon N, Urban-Mead KR, Park MG, Gibbs J, et al. Agriculturally dominated landscapes reduce bee phylogenetic diversity and pollination services. Science. 2019;363(6424):282–4.
4. Allen-Wardell G, Bernhardt P, Bitner R, Burquez A, Buchmann S, Cane J, et al. The potential consequences of pollinator declines on the conservation of biodiversity and stability of food crop yields. Conservation Biology. 1998;12(1):1–10.
5. Kremen C, Williams NM, Thorp RW. Crop pollination from native bees at risk from agricultural intensification. Proceedings of the National Academy of Sciences. 2002;99(26):16812–6.
6. Winfree R, Gross BJ, Kremen C. Valuing pollination services to agriculture. Ecological Economics. 2011;71:80–8.

7. Sánchez-Bayo F, Wyckhuys KAG. Worldwide decline of the entomofauna: A review of its drivers. Biological Conservation. 2019;232:8–27.
8. Hall D, Camilo GR, Tonietto RK, Ollerton J, Ahrné K, Arduser M, et al. The city as a refuge for insect pollinators. Conservation Biology. 2017;31(1):24–9.
9. Cardoso MC, Gonçalves RB. Reduction by half: the impact on bees of 34 years of urbanization. Urban Ecosyst. 2018;21(5):943–9.
10. Wenzel A, Grass I, Belavadi VV, Tscharntke T. How urbanization is driving pollinator diversity and pollination – A systematic review. Biological Conservation. 2020;241:108321.
11. Mcdonald RI, Kareiva P, Forman RTT. The implications of current and future urbanization for global protected areas and biodiversity conservation. Biological Conservation. 2008;141(6):1695–703.
12. Ganser D, Albrecht M, Knop E. Wildflower strips enhance wild bee reproductive success. Journal of Applied Ecology. 2021;58(3):486–95.
13. Ouvrard P, Transon J, Jacquemart AL. Flower-strip agri-environment schemes provide diverse and valuable summer flower resources for pollinating insects. Biodiversity and Conservation. 2018;1–24.
14. Hicks DM, Ouvrard P, Baldock KCR, Baude M, Goddard MA, Kunin WE, et al. Food for pollinators: quantifying the nectar and pollen resources of urban flower meadows. PLOS ONE. 2016;11(6):e0158117.
15. Schubert LF, Hellwig N, Kirmer A, Schmid-Egger C, Schmidt A, Dieker P, et al. Habitat quality and surrounding landscape structures influence wild bee occurrence in perennial wildflower strips. Basic and Applied Ecology. 20221;60:76–86.
16. Grab H, Danforth B, Poveda K, Loeb G. Landscape simplification reduces classical biological control and crop yield. Ecological Applications. 201818;28(2):348–55.
17. McCullough CT, Angelella GM, O’Rourke ME. Landscape context influences the bee conservation value of wildflower plantings. Environmental Entomology. 2021;50(4):821–31.
18. Rathcke B. Competition and facilitation among plants for pollination. In: Pollination Biology. Elsevier; 2012. p. 305–25.
19. Johnson SD, Peter CI, Nilsson LA, Ågren J. Pollination success in a deceptive orchid is enhanced by co-occurring rewarding magnet plants. Ecology. 2003;84(11):2919–27.
20. Johnson CA, Dutt P, Levine JM. Competition for pollinators destabilizes plant coexistence. Nature. 2022;607(7920):721–5.
21. Millard J, Outhwaite CL, Kinnersley R, Freeman R, Gregory RD, Adedoja O, et al. Global effects of land-use intensity on local pollinator biodiversity. Nat Commun. 2021;12(1):2902.
22. Winfree R, Bartomeus I, Cariveau DP. Native pollinators in anthropogenic habitats. Annual Review of Ecology, Evolution, and Systematics. 2011;42(1):1–22.

23. Jha S, Kremen C. Urban land use limits regional bumble bee gene flow. Molecular Ecology. 2013;22(9):2483–95.
24. Nguyen PN, Rehan SM. The effects of urban land use gradients on wild bee microbiomes. Front Microbiol. 2022;13: https://doi.org/10.3389/fmicb.2022.992660.
25. Hendrickx F, Maelfait JP, Van Wingerden W, Schweiger O, Speelmans M, Aviron S, et al. How landscape structure, land-use intensity and habitat diversity affect components of total arthropod diversity in agricultural landscapes. Journal of Applied Ecology. 2007;44(2):340–51.
26. Ayers AC, Rehan SM. Bee–plant interaction and community response along an urbanization gradient. Journal of Urban Ecology. 2023;9(1):juad006.
27. Weber M, Diekötter T, Dietzsch AC, Erler S, Greil H, Jütte T, et al. Urban wild bees benefit from flower-rich anthropogenic land use depending on bee trait and scale. Landsc Ecol. 2023;38(11):2981–99.
28. Bennett AB, Lovell S. Landscape and local site variables differentially influence pollinators and pollination services in urban agricultural sites. PLOS ONE. 2019;14(2):e0212034.
29. Williams NM, Ward KL, Pope N, Isaacs R, Wilson J, May EA, et al. Native wildflower plantings support wild bee abundance and diversity in agricultural landscapes across the United States. Ecological Applications. 2015;25(8):2119–31.
30. Russo L, Vaudo AD, Fisher CJ, Grozinger CM, Shea K. Bee community preference for an invasive thistle associated with higher pollen protein content. Oecologia. 2019;190(4):901–12.
31. Russo L, Buckley YM, Hamilton H, Kavanagh M, Stout JC. Low concentrations of fertilizer and herbicide alter plant growth and interactions with flower-visiting insects. Agriculture, Ecosystems and Environment. 2020;304:107141.
32. Laliberté E, Norton DA, Tylianakis JM, Scott D. Comparison of two sampling methods for quantifying changes in vegetation composition under rangeland development. Rangeland Ecology & Management. 2010;63(5):537–45.
33. Ascher J, Pickering J. Discover life: bee species guide and world checklist (Hymenoptera: Apoidea: Anthophila). 2017.
34. Khalil A, Sykes V, Russo L. Floral identity and availability along with surrounding landscapes affect pollinator communities in eastern Tennessee. Landsc Ecol. 2023;38(10):2623–38.
35. Zurbuchen A, Landert L, Klaiber J, Müller A, Hein S, Dorn S. Maximum foraging ranges in solitary bees: only few individuals have the capability to cover long foraging distances. Biological Conservation. 2010 Mar;143(3):669–76.
36. Redhead JW, Dreier S, Bourke AFG, Heard MS, Jordan WC, Sumner S, et al. Effects of habitat composition and landscape structure on worker foraging distances of five bumble bee species. Ecological Applications. 2016;26(3):726–39.

37. R Core Team. R: A language and environment for statistical computing. R Foundation for Statistical Computing, Vienna, Austria. 2021; Available from: http://www.r-project.org/
38. Bates D, Mächler M, Bolker B, Walker S. Fitting linear mixed-effects models using lme4. Journal of Statistical Software. 2014;67(1):1–48.
39. Oksanen J, Blanchet G, Kindt R, Legendre P, O'Hara RB, Simpson G, et al. Vegan: Community ecology package, 2011. R Package Version. 2011;1–17.
40. De Cáceres M, Legendre P. Associations between species and groups of sites: indices and statistical inference. Ecology. 2009;90(12):3566–74.
41. Carvalheiro LG, Veldtman R, Shenkute AG, Tesfay GB, Pirk CWW, Donaldson JS, et al. Natural and within-farmland biodiversity enhances crop productivity. Ecology letters. 2011;14(3):251–9.
42. Kammerer MA, Biddinger DJ, Rajotte EG, Mortensen DA. Local plant diversity across multiple habitats supports a diverse wild bee community in Pennsylvania apple orchards. Environmental Entomology. 2016;45(1):32–8.
43. Karron JD, Mitchell RJ. Effects of floral display size on male and female reproductive success in Mimulus ringens. Annals of Botany. 2012;109(3):563–70.
44. Schoch K, Tschumi M, Lutter S, Ramseier H, Zingg S. Competition and facilitation effects of semi-natural habitats drive total insect and pollinator abundance in flower strips. Frontiers in Ecology and Evolution. 2022;10. Available from: https://www.frontiersin.org/articles/10.3389/fevo.2022.854058
45. Winfree R, Aguilar R, Vázquez DP, LeBuhn G, Aizen MA. A meta-analysis of bees' responses to anthropogenic disturbance. Ecology. 2009;90(8):2068–76.
46. Ruiz-Toledo J, Vandame R, Penilla-Navarro P, Gómez J, Sánchez D. Seasonal abundance and diversity of native bees in a patchy agricultural landscape in Southern Mexico. Agriculture, Ecosystems & Environment. 2020 15;292:106807.
47. Ekroos J, Heliölä J, Kuussaari M. Homogenization of lepidopteran communities in intensively cultivated agricultural landscapes. Journal of Applied Ecology. 2010;47(2):459–67.
48. Dormann CF, Schweiger O, Augenstein I, Bailey D, Billeter R, De Blust G, et al. Effects of landscape structure and land-use intensity on similarity of plant and animal communities. Global Ecology and Biogeography. 2007;16(6):774–87.
49. Wignall VR, Brolly M, Uthoff C, Norton KE, Chipperfield HM, Balfour NJ, et al. Exploitative competition and displacement mediated by eusocial bees: experimental evidence in a wild pollinator community. Behavioral Ecology and Sociobiology. 2020;74(12):1–15.
50. Rasmussen C, Dupont YL, Madsen HB, Bogusch P, Goulson D, Herbertsson L, et al. Evaluating competition for forage plants between honey bees and wild bees in Denmark. PLOS ONE. 2021;16(4):e0250056.
51. M'Gonigle LK, Ponisio LC, Cutler K, Kremen C. Habitat restoration promotes pollinator persistence and colonization in intensively managed agriculture. Ecological Applications. 2015;25(6):1557–65.

Table 1. Results from generalized linear mixed effects models (GLMMs) for bee abundance and species richness in the gardens and local area. Significant effects are bolded. When the residuals were not normal, the responses were log-transformed and we used a Gaussian distribution for all models. The marginal R^2 reflects the percent variation explained by the fixed effects in the model and the conditional R^2 reflects the percent variation explained by the full model (random and fixed effects).

**Response**	**Fixed effects**	**Contrasts**	**Random effects**	**Observations**	**Effect size**	**t value**	**P value**	**R^2m**	**R^2c**
Garden Abundance (log)	**Garden Flowers**	continuous	Round|Plot	100 obs, 4 plot types, 4 rounds	**1.51**	2.98	**0.003**	0.30	0.51
	Local Flowers				0.33	0.49	0.62		
	**Garden Diversity**				**1.76**	2.93	**0.003**		
	**Local Diversity**				**-1.48**	-2.05	**0.04**		
	**Development (2km)**				**0.02**	2.89	**0.004**		
Local Abundance (log)	**Local Flowers**	continuous	Round	100 obs, 4 rounds	**2.72**	3.83	**<0.001**	0.40	0.42
	Local Diversity				-0.94	-1.21	0.23		
	Development (2km)				0.01	1.82	0.07		
Garden Species Richness	**Garden Flowers**	continuous	Round|Plot	100 obs, 4 plot types, 4 rounds	**5.74**	4.31	**<0.001**	0.29	0.44
	Garden Diversity				2.3	1.45	0.15		
	Local Flowers				0.08	0.04	0.97		
	Local Diversity				-1.79	-0.91	0.36		
	**Development (2km)**				**0.04**	2.04	**0.04**		
Local Species Richness	**Local Flowers**	continuous	Round	100 obs, 4 rounds	**5.11**	2.14	**0.03**	0.31	0.36
	Local Diversity				1.23	0.47	0.64		
	Development (2km)				0.02	0.69	0.49		

FIGURES

Figure 1.

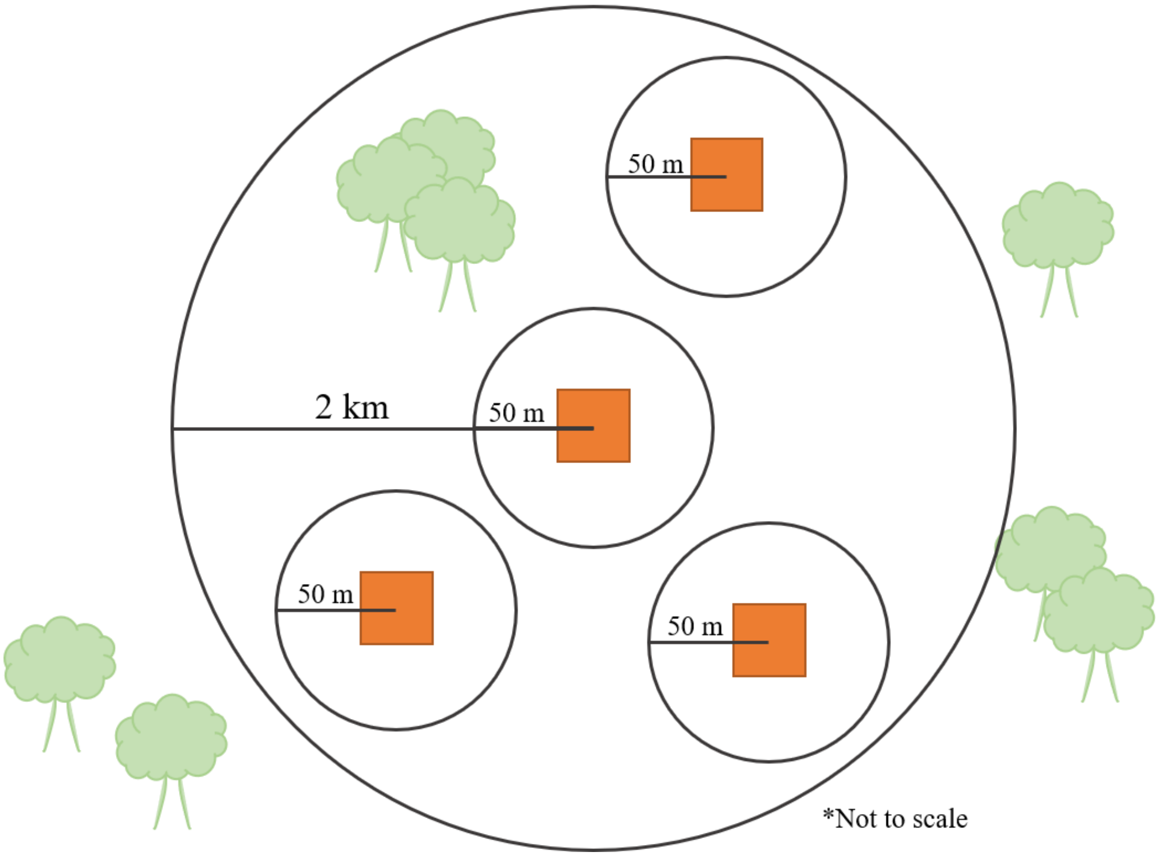

Figure 1. A heuristic diagram of the sampling design for a single site. The four research gardens are represented by orange squares which are surrounded by the 50-meter local sampling area. The 2-kilometer circle around all four gardens represents the area included in the GIS land-use analysis.

Figure 2.

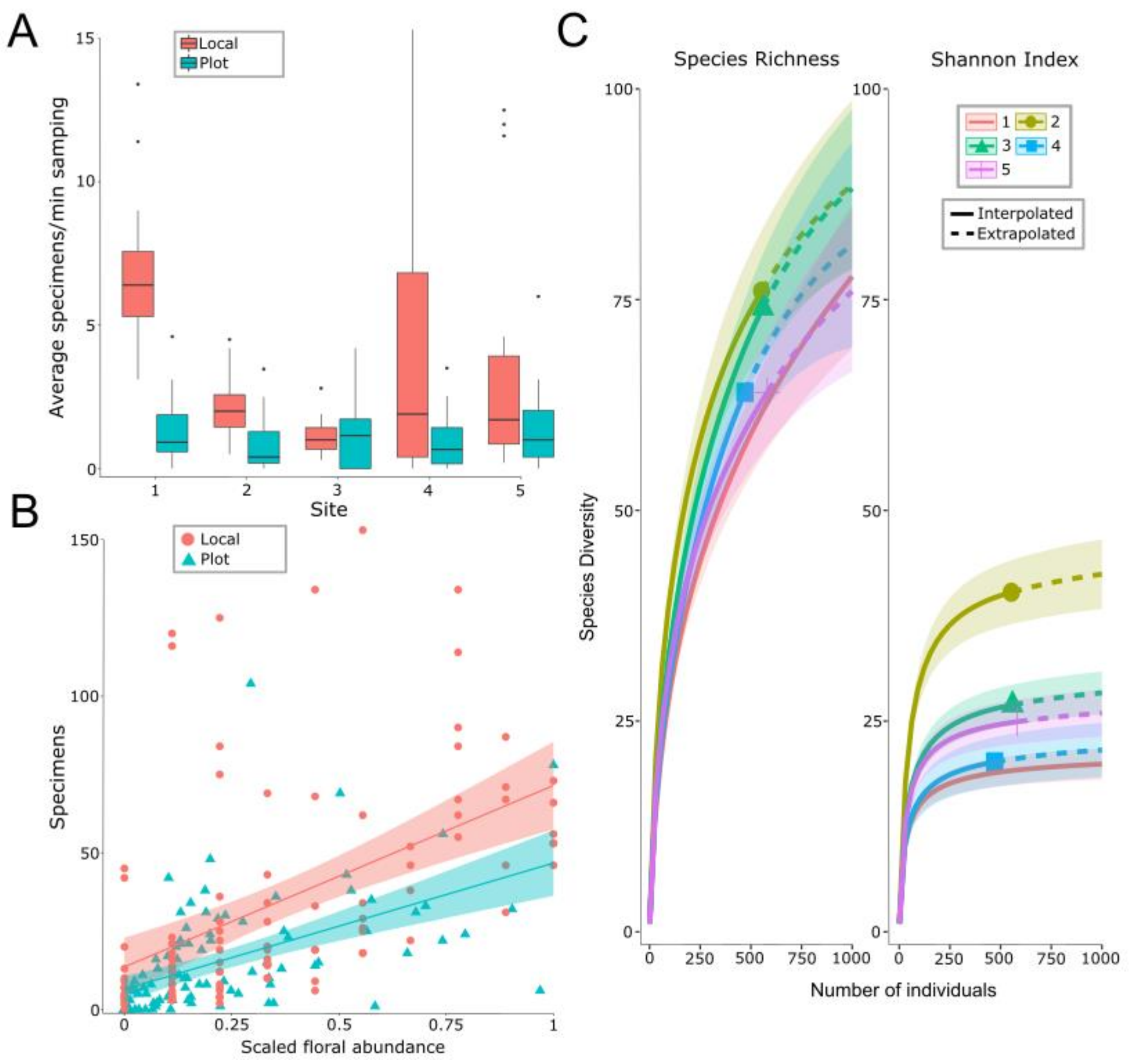

Figure 2. A) Box and whisker plots of the average number of bees collected per minute sampled at all five sites (1: Urban Garden, 2: Forage Grassland, 3: Mixed Agriculture, 4: Forest, and 5: Organic Farm) for the landscape (red) and plot (blue) surveys. B) Number of bees collected per sample compared to the scaled floral display of the local (red) and garden (blue) surveys. The shaded area around each line is the 95% confidence interval. C) Rarefaction analysis (excluding honeybees) showing bee species richness and

Shannon diversity index at the five sites (1 = red, 2 = yellow, 3 = green, 4 = blue, 5 = purple). Interpolated data are represented with a solid line and extrapolated data are represented with a dashed line.

Figure 3.

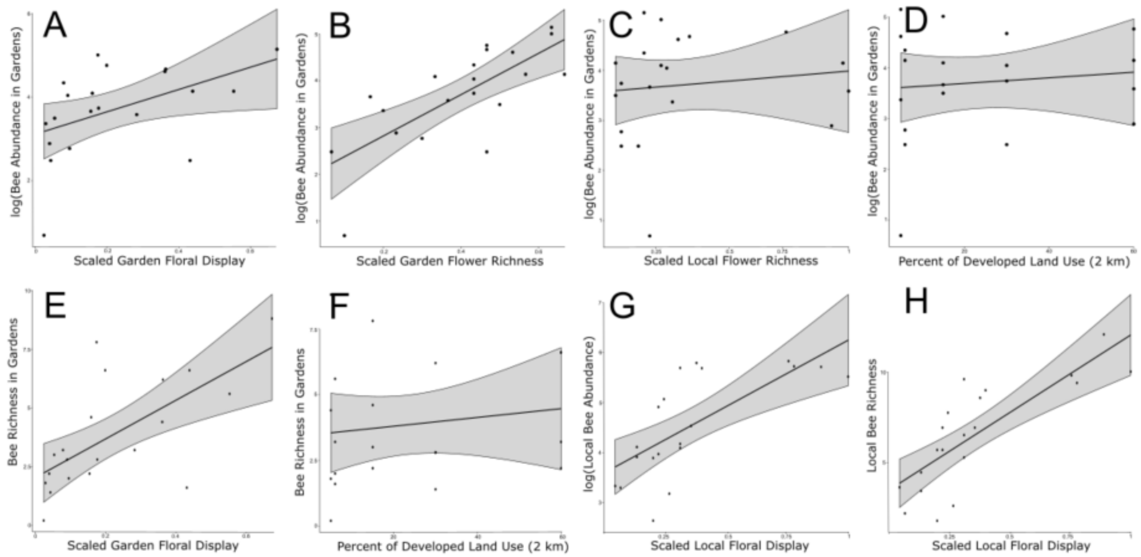

Figure 3. Significant linear relationships between bee abundance and species richness in the gardens and local area. We found that bee abundance in the gardens was driven most strongly by the floral display (A) and flower richness (B) in the gardens, and to a lesser extent by local flower richness (C) and developed land use in a 2 km radius (D). Bee richness in the gardens was driven mostly by the floral display in the gardens (E), and to a lesser extent by the percent of developed land use in a 2 km radius (F). The local bee abundance (G) and richness (H) were mainly driven by the local floral display.

Figure 4.

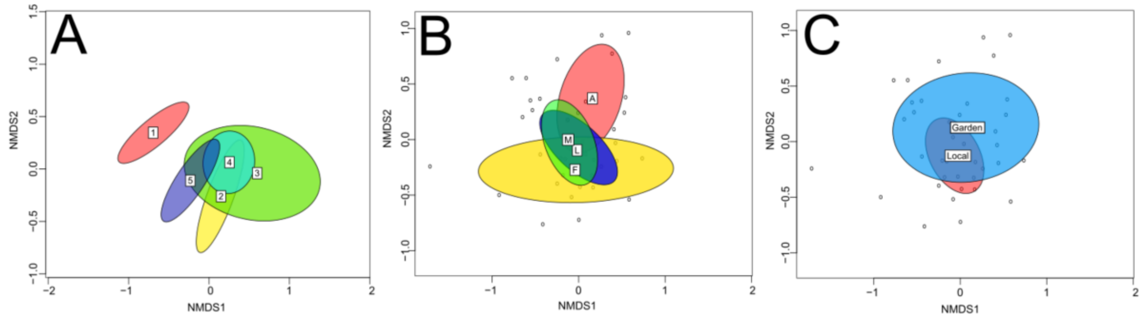

Figure 4. Non-metric multidimensional scaling (NMDS) ordination plots of the different sites (A), plot types (B), and surveys (C). Overlap in the shapes indicates overlap in the communities in the sites or surveys. Color indicates landscape (A): Urban Garden (red), Forage Grassland (yellow), Mixed Agriculture (green), Forest (light blue), and Organic Farm (dark blue); plot type: Lamiaceae (dark blue), Fabaceae (yellow), Asteraceae (red), and Mixed (green); and survey type: garden (blue) and local (red).

Click here to access/download;Figure;Eldridgeetal_Fig3.pdf

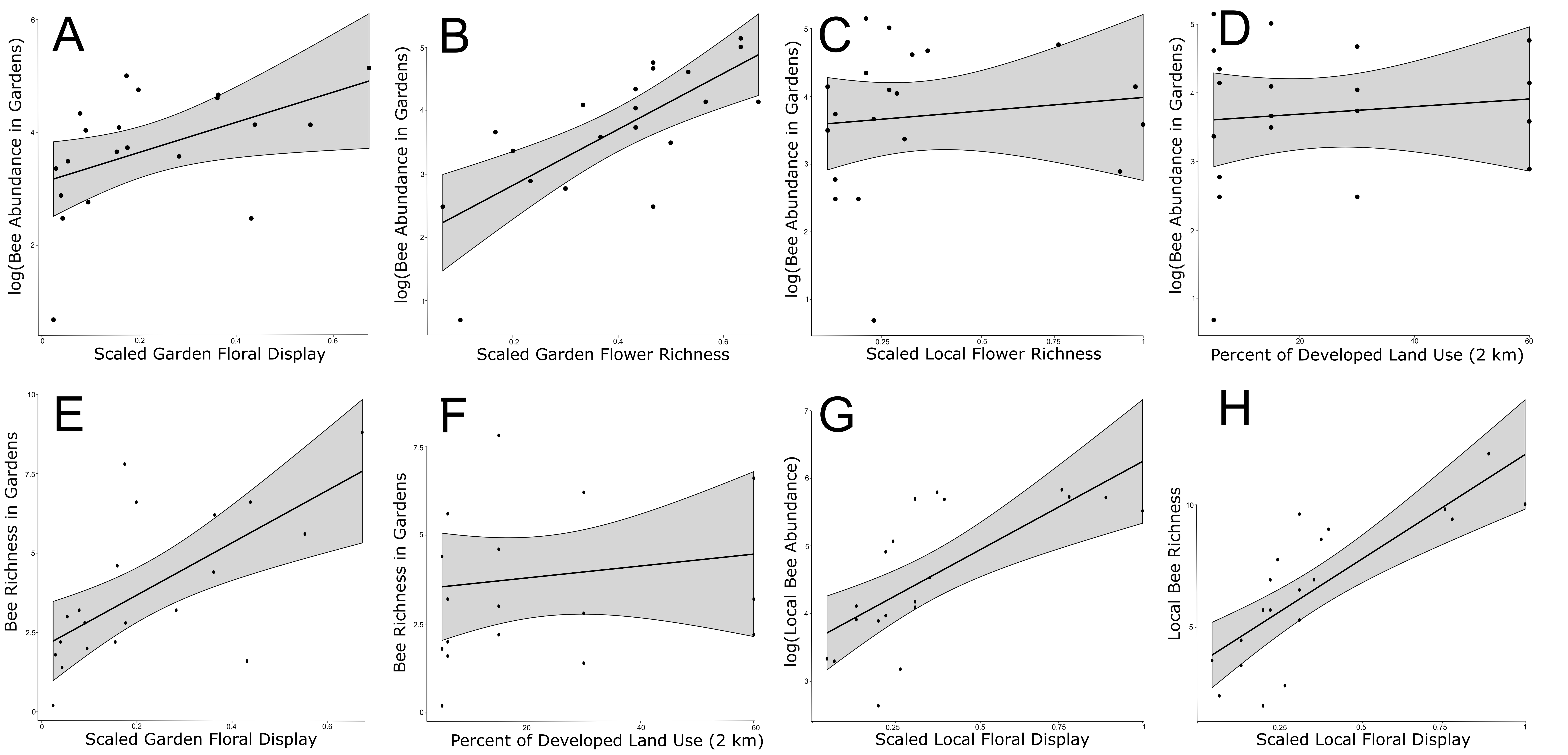

Click here to access/download;Figure;Eldridgeetal_Fig2.pdf

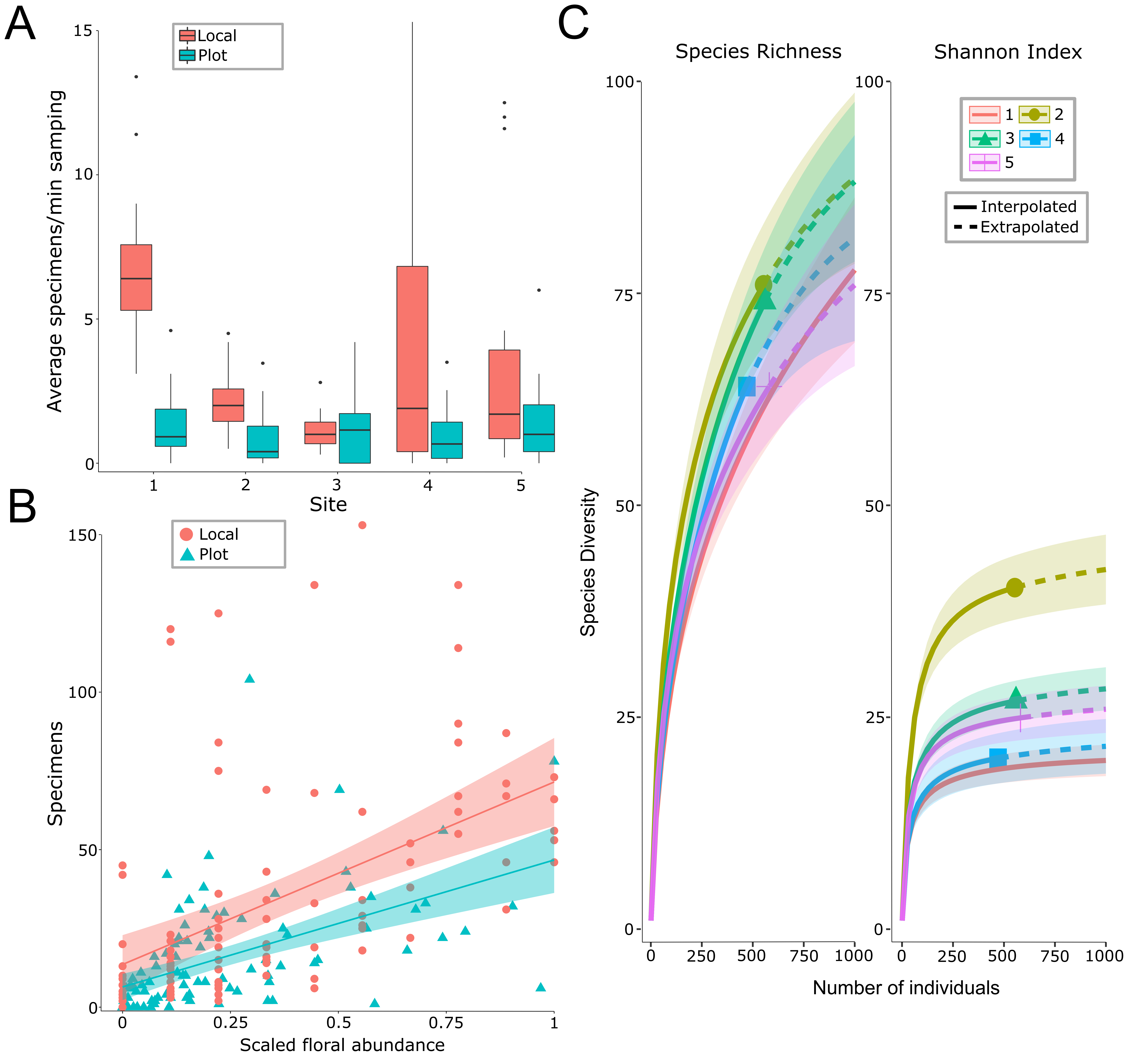

Click here to access/download;Figure;Eldridgeetal_Fig4.pdf

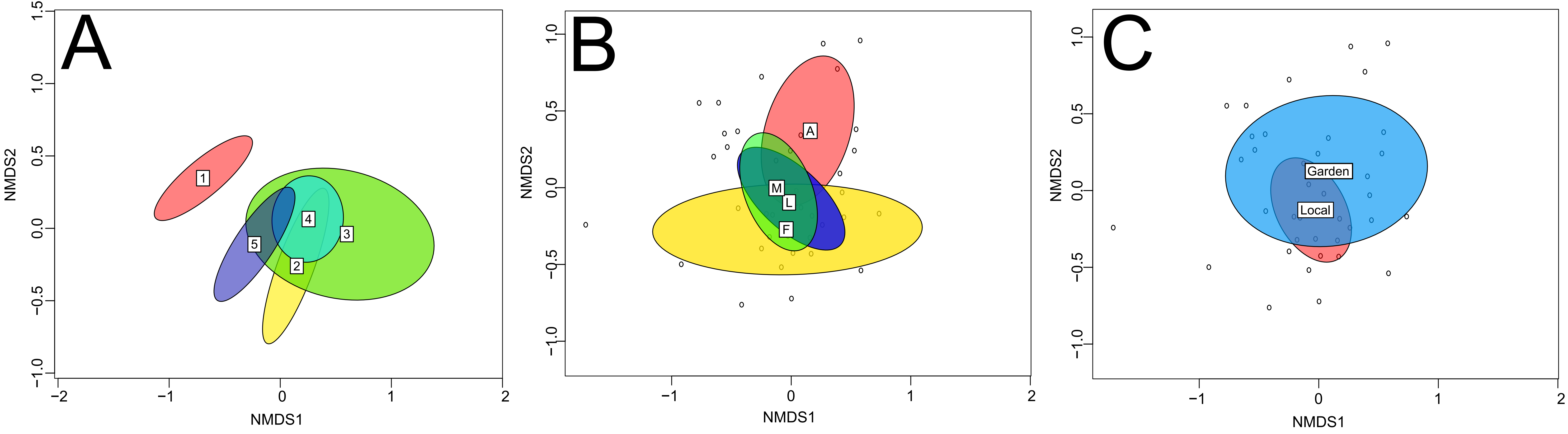

Click here to access/download
Supporting Information
Eldridgeetal_SupportingInformation_PLoSONE.docx

Click here to access/download
Supporting Information
Eldridgeetal_SupportingInformation_2.xlsx
