# Supplementary material for: Do local and landscape context affect the attractiveness of flower gardens to bees?
Source: PLoS One. 2024 Sep 4;19(9):e0309000. doi: 10.1371/journal.pone.0309000 (PMC11373812; doi:10.1371/journal.pone.0309000)
Supplement: Attachment — Submitted filename: Response to Reviewers.docx [file pone.0309000.s004.docx]

PONE-D-24-09528R1

Do local and landscape context affect the attractiveness of flower gardens to bees?

PLOS ONE

Dear Dr. Russo,

Thank you for submitting your manuscript to PLOS ONE. After careful consideration, we feel that it has merit but does not fully meet PLOS ONE’s publication criteria as it currently stands. Therefore, we invite you to submit a revised version of the manuscript that addresses the points raised during the review process.

Please submit your revised manuscript by Aug 25 2024 11:59PM. If you will need more time than this to complete your revisions, please reply to this message or contact the journal office at [plosone@plos.org](mailto:plosone@plos.org). When you're ready to submit your revision, log on to <https://www.editorialmanager.com/pone/> and select the 'Submissions Needing Revision' folder to locate your manuscript file.

Please include the following items when submitting your revised manuscript:

- A rebuttal letter that responds to each point raised by the academic editor and reviewer(s). You should upload this letter as a separate file labeled 'Response to Reviewers'.
- A marked-up copy of your manuscript that highlights changes made to the original version. You should upload this as a separate file labeled 'Revised Manuscript with Track Changes'.
- An unmarked version of your revised paper without tracked changes. You should upload this as a separate file labeled 'Manuscript'.

If you would like to make changes to your financial disclosure, please include your updated statement in your cover letter. Guidelines for resubmitting your figure files are available below the reviewer comments at the end of this letter.

If applicable, we recommend that you deposit your laboratory protocols in [protocols.io](http://protocols.io) to enhance the reproducibility of your results. Protocols.io assigns your protocol its own identifier (DOI) so that it can be cited independently in the future. For instructions see: <https://journals.plos.org/plosone/s/submission-guidelines#loc-laboratory-protocols>. Additionally, PLOS ONE offers an option for publishing peer-reviewed Lab Protocol articles, which describe protocols hosted on [protocols.io](http://protocols.io). Read more information on sharing protocols at <https://plos.org/protocols?utm_medium=editorial-email&utm_source=authorletters&utm_campaign=protocols>.

We look forward to receiving your revised manuscript.

Kind regards,

Dr. Janice L. Bossart
Academic Editor
PLOS ONE

***We would like to thank the editors for the helpful comments. We have addressed all the comments on a point by point basis below, demarcated with three asterisks.***

Journal Requirements:

Please review your reference list to ensure that it is complete and correct. If you have cited papers that have been retracted, please include the rationale for doing so in the manuscript text, or remove these references and replace them with relevant current references. Any changes to the reference list should be mentioned in the rebuttal letter that accompanies your revised manuscript. If you need to cite a retracted article, indicate the article’s retracted status in the References list and also include a citation and full reference for the retraction notice.

**Additional Editor Comments:**

The revised version has corrected most of the problems identified by reviewers.  Thank you for your efforts to address their concerns.  Below I list remaining issues that need attention.

**Thank you for these helpful comments!***

Line 124.  Missing 'the'.  Three of the...

***Fixed.***

Line 439.  Missing word.  There 'was' ?

***Fixed.***

Line 728.  Please spell out linear model(s) so it's not confused with 1m, and either make model plural or add an 'a' before linear.

***We changed this to “The lines are drawn with the function “geom_smooth(method = lm)” in the package *ggplot2*...” to clarify this sentence.***

Table 1.  Delete the extraneous 's' at the end.

***Fixed.***

Figure S1.  The legend struck me as a bit odd given 'Bees' aren't an Order.  Maybe Hymenoptera-Bees and Hymenoptera-Non Bees?  Or maybe you can think of some other way that all terms fit.

***We changed “Order” to “Taxa” in this figure to correct for the different categories.***

Figure 3C.  Given the overlap of confidence intervals of 'Mixed' and at least 'Organic', I'm not sure I buy the interpretation that both 'Forage' and 'Mixed' had higher Shannon Diversity than all others.

***For the supplemental figure showing the results of the rarefaction analyses, we realized it should be labeled as Figure S2, instead of S3, so we corrected that. The Forage and Mixed had higher Shannon Diversity, but only when honeybees are included, whereas the figure in the main text shows the honeybees removed, so we clarified this in text. We also added a new supplemental table with the raw data output from the rarefaction analysis showing the estimated diversity and its confidence intervals, and the sample coverage for the sites and surveys.***

--Mismatch between Figure 3 and Figure 3 caption.  Letter designations in the Figure caption don't match letter designations associated with plots in the actual figure.  Also, there is no plot of local bee abundance and land development (I'm guessing it's the caption that's incorrect given what is stated in the Results section).

***Thank you for catching that! We corrected the figure caption to match the figure.***

--Mismatch between Table 1 and text in the Methods.  Is sampling round nested within Site or nested within Plot?  The Table says Plot, the text says Site (Line 273).  Regardless of which is correct, the text would read better if you delete 'identity'.

***The table was correct, so we modified the main text to match. We deleted “identity” in the text.***

--Loose use of 'diversity' throughout.  Given diversity can be used in multiple ways, please don't use it when you are referring specifically to species richness of either bees or flowers.  Otherwise, readers can't be sure whether you mean diversity, in terms of both richness and evenness, or simply richness.  Please carefully go through the manuscript, including figures and tables, and replace your use of 'diversity' with 'richness' anywhere you are explicitly referring to richness.

***Thank you for this, we have revised the text throughout, and checked the figures in the main text and supplement. We include “diversity” rather than “species richness” under the following circumstances: 1) for bee diversity when we are referring both to species richness and Shannon diversity, 2) for the plant diversity where we ranked floral diversity on a scale of 1-10 rather than quantifying species richness of the flowering plants. In addition to these edits, we reviewed our use of floral display throughout to make it more consistent.***

--Be consistent across Text, Figures, Tables, etc. including supplemental.  For example, Figure S2 uses Shannon Diversity and Figure 2C uses Shannon Index.  I didn't check every use, but Shannon diversity is often used in the text so perhaps that would be the way to go throughout.  Also, Figure S2C is unnecessary given it is identical to Figure 2C.

***We changed Shannon Index to Shannon Diversity throughout and checked the figures as well. We agree that is the more appropriate term. We removed Figure S2C (it was replaced with sample coverage graphs).***

--Rarefaction & Coverage methods. There is insufficient information in this section of the manuscript, e.g. approx. 256-267, to understand data shown in the resultant Figures.  iNEXT calculates Hill numbers, which at least needs to be pointed out as these are different calculations than other methods use.  Also, your figures don't correspond with figures generated by iNEXT for sample coverage or completeness, e.g. I'm expecting coverage or completeness to be an axis label.  No doubt I'm missing something, but if I am then others will likely also be confused.  Please add a bit of text to clarify how coverage was estimated and depicted.

***Thank you for pointing out this omission. We added detail about the methods used (and Hill numbers) to the methods. We also add new graphs to the supplemental figure to show the sampling completeness of the surveys and sites.***

[Note: HTML markup is below. Please do not edit.]

[NOTE: If reviewer comments were submitted as an attachment file, they will be attached to this email and accessible via the submission site. Please log into your account, locate the manuscript record, and check for the action link "View Attachments". If this link does not appear, there are no attachment files.]

While revising your submission, please upload your figure files to the Preflight Analysis and Conversion Engine (PACE) digital diagnostic tool, <https://pacev2.apexcovantage.com/>. PACE helps ensure that figures meet PLOS requirements. To use PACE, you must first register as a user. Registration is free. Then, login and navigate to the UPLOAD tab, where you will find detailed instructions on how to use the tool. If you encounter any issues or have any questions when using PACE, please email PLOS at [figures@plos.org](mailto:figures@plos.org). Please note that Supporting Information files do not need this step.
